# Advance care planning by proxy for residential aged care facility residents: results of a pilot interventional study

**DOI:** 10.1093/geroni/igag033

**Published:** 2026-04-07

**Authors:** Laura Jones, Eve Rubli Truchard, Anca-Cristina Sterie, Yaëlle Rhyner, Elisabeth Iorio, Ralf J Jox

**Affiliations:** Chair of Geriatric Palliative Care, Lausanne University Hospital and University of Lausanne, Lausanne, Switzerland; Chair of Geriatric Palliative Care, Lausanne University Hospital and University of Lausanne, Lausanne, Switzerland; Department of Geriatrics and Rehabilitation, Lausanne University Hospital and University of Lausanne, Lausanne, Switzerland; Chair of Geriatric Palliative Care, Lausanne University Hospital and University of Lausanne, Lausanne, Switzerland; Service of Palliative and Supportive Care, Lausanne University Hospital and University of Lausanne, Lausanne, Switzerland; Centre Senior, Ensemble Hospitalier de la Côte, Morges Hospital, Morges, Switzerland; Service of Clinical Practice Development, Ensemble Hospitalier de la Côte, Morges Hospital, Morges, Switzerland; Chair of Geriatric Palliative Care, Lausanne University Hospital and University of Lausanne, Lausanne, Switzerland; Institute of Humanities in Medicine, Lausanne University Hospital and University of Lausanne, Lausanne, Switzerland

**Keywords:** Dementia, Decision-making, Long-term care, Person-centered care, Institutional care/residential care

## Abstract

**Background and Objectives:**

Advance care planning (ACP) allows people with decision-making capacity (DMC) to stipulate their wishes for medical treatment if they lose this capacity. However, many lose DMC before expressing their treatment wishes. Supplementary ACP models, conducted with health care proxies (HCPs), could promote care that is as coherent as possible with the presumed wishes of people who have lost medical DMC. This study pilot-tested an ACP-by proxy (ACP-bp) intervention with HCPs of residential aged care facility (RACF) residents lacking medical DMC to examine the acceptability and feasibility of the intervention.

**Research Design and Methods:**

21 HCPs of 20 RACF residents participated in this multi-method evaluation of a single-arm intervention pilot study. Proxy decisional conflict and facilitator (RACF health professionals) self-efficacy were measured pre- and post-intervention. Semi-structured interviews were conducted with HCPs and facilitators. Interviews were audio-recorded, transcribed verbatim, and analyzed thematically.

**Results:**

The intervention significantly reduced proxy decisional conflict. Feasibility was threatened by health professional, proxy, and physician time, and RACF health professional turnover. Proxies and facilitators indicated that ACP-bp promoted in-depth discussions of residents’ lives, thereby improving health professionals’ knowledge and connection with residents, as well as proxy confidence. It promoted communication and cooperation between proxies and health professionals and provided clear documentation in case of emergency.

**Discussion and Implications:**

The intervention was acceptable for all participants. Future research should focus on the most efficient models for implementing ACP-bp and establish their validity in promoting care that is coherent with patient wishes through a cluster randomized control study.

**Clinical Trial Registration Number:**

NCT04779684

Innovation and Translational Significance: We developed and evaluated the acceptability and feasibility of an innovative model of Advance Care Planning-by proxy (ACP-bp) in residential aged care facility (RACF) settings. The intervention was highly accepted and significantly reduced health care proxy decisional conflict. ACP-bp presents an innovative opportunity to promote care that is coherent with the wishes of people who no longer have medical decision-making capacity, therefore promoting their autonomy, reducing distress for their entourage, improving professional-proxy communication, and directing health resources toward goal-concordant care. Our findings provide insights into the factors necessary to implement ACP-bp soon after RACF admission.

## Background and objectives

Global population aging is a historically unprecedented challenge that is profoundly transforming health care. One major implication is the increasing number of people with impaired decision-making capacity due to age-related neuropsychiatric disorders, notably dementia (Lepping et al., 2015). For most forms of dementia, the persons affected lose their decision-making capacity for major treatment and care decisions relatively early during the disease trajectory (Hamann et al., 2011). This means that they have lost decision-making capacity when major treatment decisions are necessary, especially regarding life-sustaining treatment decisions in the advanced stages (e.g., life-sustaining treatment of pneumonia). There is a general consensus that patient autonomy should be respected as much as possible in these situations. Yet, involving people who have lost decision-making capacity in treatment decisions and establishing their presumed treatment wishes is complex. This complexity threatens their self-determination (Morris et al., 2021).

If patients are evaluated as incapable of making their health care decisions and applicable advance directives are not present, their health care proxies are called upon to make decisions based on substituted judgment—i.e., exploring the presumed preferences of the persons, or according to the latter’s best interests. Current verbal and non-verbal communication, previous statements and documents, expression of values and life attitudes form the basis of this decision-making (in der Schmitten et al., 2021). However, in regard to treatment decisions, proxy decision making often fails to represent the patient’s wishes (Shalowitz et al., 2006). Moreover, it can lead to significant proxy distress and decisional conflict, especially when it comes to planning care or making primary care treatment decisions in geriatrics (Garvelink et al., 2019).

Advance care planning (ACP) is a complex health care intervention that allows people with decision-making capacity to extend their medical decision-making autonomy to situations in which they no longer have the capacity to make their own decisions. This occurs through a facilitated process in which they reflect on their values and wishes for future medical care, name a health care proxy, document their wishes for medical care, and review them when circumstances change (Rietjens et al., 2017). Multiple systematic reviews have shown that ACP increases the likelihood that incapacitated patients are treated according to their wishes (Dixon et al., 2018). It also reduces proxy distress and morbidity (Detering et al., 2010).

Maintaining decision-making capacity is essential for participating in traditional models of ACP (Rietjens et al., 2017). However, even in countries where ACP is well established, such as the United States and Australia, less than one-third of the population complete advance directives (Detering et al., 2019), thus suggesting that many people lose decision-making capacity before having formally documented or discussed their wishes. In addition, even when people know that they will lose decision-making capacity in the near future, participation in ACP is plagued by many obstacles (Bosisio et al., 2021). As compared to short-term loss of decision-making capacity (for example in the case of an acute brain injury from an accident or delirium), ACP is more complicated in the case of prolonged loss of decision-making capacity, such as dementia: many changes in the clinical situation may occur, including personality transformation, and proxies may need or wish to revise existing or complete new ACPs on behalf of the incapacitated person (Cohen & DeMartino, 2021; Jox, 2016). In countries such as Switzerland, where ACP is relatively new (Clavien et al., 2023), people are more likely to have completed a simple, check-box type Advance Directive (AD) than to have participated in comprehensive ACP discussions. Yet, in Switzerland, AD completion is also low, with less than half of older adults (58+ years) completing an AD or appointing a health care proxy (Meier et al., 2024). Even when ADs have been completed, there are many barriers to their implementation, related to the fact that these documents can be completed individually, and no accessible register exists, which undermines their usefulness in practice (Baumann et al., 2023). ADs are only one part of in-the-moment proxy decision making and typically lack the essential features of discussing values and quality of life, communication about proxy identification, their roles and decision leeway, and informing family of plans (Fagundes et al., 2021; in der Schmitten et al., 2021; McMahan et al., 2013; Merche et al., 2025; Tewari et al., 2025).

These considerations highlight the need for Advance Care Planning by proxy (ACP-bp) in order to promote self-determination of people who have lost medical decision-making capacity. This model has been conceptually suggested by Volicer and colleagues (2002) and later has been recommended for all individuals who have lost decision-making capacity by in der Schmitten et al. (2021). However, with the exception of anecdotal practice reports and one intervention study focused on family conferences (Brazil et al., 2018), ACP-bp has not yet been systematically developed. ACP programs such as Respecting Choices also involve proxies of incapacitated persons, but they rarely have specific approaches or processes for ACP-bp, and their evidence base relies on traditional ACP models (designed for people who maintain decision-making capacity) (Coors & Jox, 2015). The importance of a facilitated process for conducting ACP with family members of people with advanced dementia has been identified in a systematic review (Chan & Woo, 2025). Yet, it is essential to differentiate ACP-bp from traditional ACP models because its ethico-legal basis is substantially different, as are the social role and the psychological involvement of proxies (Jox, 2016). ACP-bp is driven by proxies who reconstruct the patient’s presumed preferences, apply them to anticipated medical situations in a dialogue with health care professionals, and assume legal responsibility for these advance decisions, which may or may not be documented in specific “surrogate directives” (Jox, 2019). ACP-bp raises the difficult question of how to involve the affected persons themselves despite their lack of medical decision-making capacity, seeking informed assent. The ethical justification for ACP-bp models, and principles for guiding the development of such models (in der Schmitten et al., 2021), can guide the development of ACP-bp models.

The period where complex care decisions must be made in the absence of decision-making capacity for people with moderate or advanced dementia creates an urgent need for ACP-bp. People with dementia report avoiding medical and end-of-life care planning in the early stages of dementia, instead focusing on post-death affairs such as wills and funeral arrangements (Poole et al., 2018). This lack of care planning has been attributed to prognostic uncertainty, communication difficulties, and the ethical complexity of treatment decisions throughout the course of dementia (Bosisio et al., 2018). Among people with dementia, older age and advanced stages of dementia are associated with increased ACP participation (Zhai et al., 2024), but generally, persons with dementia engage in ACP at lower rates than the general population (Blake et al., 2017; Dening et al., 2012), which is one of the reasons for high rates of unwanted or even medically inappropriate interventions in the advanced stages of dementia, particularly at the end of life (Gozalo et al., 2011). Previous research has highlighted the need for ACP interventions for late-stage dementia (Goldstein & Mather, 2018).

Dementia prevalence in Residential Aged Care Facility (RACF) settings is highly variable, depending on the country (range: 11%–84%, overall prevalence 54%) (Fagundes et al., 2021), while dementia is also underdiagnosed in the RACF setting to various degrees (14%–70%) (Tewari et al., 2025). In Switzerland, where home care is highly developed, and people remain at home until very advanced ages, people enter RACFs in increasingly fragile health states near the end of their lives, and the majority remain there until their death (Hedinger et al., 2014), thus necessitating complex end-of-life care decisions during their stays. The RACF context thus requires models of ACP-bp for planning and to avoid unnecessary hospitalizations that are estimated at 14%–22% (Merche et al., 2025). Analysis of AD written by proxies of German RACF residents lacking decision-making capacity has shown significant variation in their content and form, and therefore applicability, which reflects the lack of national and international guidelines for such forms (in der Schmitten et al., 2021), and further highlights the need for standardized ACP-bp processes and models. Furthermore, the need for robust research looking at the outcomes of ACP interventions for RACF residents, their families, and health professionals has been noted (Parker, 2010).

We conducted an exploratory study into the current practices and needs for ACP for people lacking medical decision-making capacity living in RACFs, which indicated the need for a structured model of planning that includes health professional training to introduce and discuss ACP-bp, discussion guides, and specific documentation (Jones et al., 2025). These results were used to develop a model of ACP-bp specifically for this context. Here we present the results of the pilot study, which aimed to assess the acceptability and feasibility of the intervention, from the perspectives of health care proxies and ACP-bp facilitators. Furthermore, given the previous literature suggesting that health care proxies experience decisional and moral distress, and previous literature suggesting the suitability of decisional conflict as an outcome measure (Brazil et al., 2018), we aimed to explore the impact of this intervention on health care proxy decisional conflict and the potential usefulness of this construct for future efficacy studies. In addition, given the physical proximity of the ACP-bp facilitators to the residents (health professionals working in the RACFs), and their role in implementing care decisions (Jones et al., 2024), we aimed to assess the impact of the intervention on their self-efficacy in treating the residents in accordance with their presumed will.

## Research design and methods

### Intervention

The ACP-bp intervention was developed in accordance with the Medical Research Council guidelines for developing complex interventions (Craig et al., 2013) comprising five steps: (1) identifying existing evidence, (2) developing theory, (3) modeling processes and outcomes, (4) assessing feasibility, and (5) evaluating the effectiveness of the intervention. Steps 1 and 2 were conducted through a qualitative exploratory study (Jones et al., 2024, 2025). Steps 3 and 4 were conducted through the present pilot study.

The ACP-bp intervention involved multiple (2–3) facilitated discussions between health care proxies of RACF residents who lack decision-making capacity (as judged by their treating physician), a RACF health professional (called “ACP-bp facilitator”) who was trained specifically for this intervention pilot, and the treating physician. The residents’ involvement in these discussions depended on their preference and capacity for involvement. First, ACP-bp facilitators (who were usually the residents’ referent carer or a social worker who knew the resident) and proxies discussed whether they thought that the resident should be invited to participate in these discussions (whether they would be able to follow and contribute to the discussion or whether the discussion was likely to provoke fatigue, confusion, or distress). If the proxies and health professionals deemed this appropriate, the intervention and research were explained to the residents, and they were asked whether they would like to be present for these discussions.

The structured ACP-bp discussions were guided by a paper discussion guide (Supplementary Material) and followed three steps: (1) identifying and reconstructing the core values of the resident; (2) anticipating probable future scenarios, typical for the trajectory of dementia, in which important treatment and care decisions would be required, especially those that would have to be made urgently; and (3) making anticipatory decisions about goals of care and treatments for specific situations based on the resident’s presumed will. Anticipatory decisions made during this process were documented in a standardized way by the facilitator on a standardized form (Supplementary Material) and signed by the health care proxy, facilitator, and treating physician.

### Inclusion criteria

#### Residents (n = 20)

1. living in one of the participating RACFs;
2. having lost decision-making capacity for major health care decisions, according to medical judgment; and
3. having a diagnosis of dementia or another cognitive disorder.

#### Health care proxies

1. legally authorized health care proxy for one of the aforementioned residents and
2. having sufficient French language proficiency to complete a written questionnaire and participate in discussions.

#### ACP-bp facilitators

1. be a health care professional with at least 12 months of RACF work experience in Switzerland and
2. be involved in daily care or care planning for residents without medical decision-making capacity.

### Recruitment, intervention, and research process

**Figure 1:**
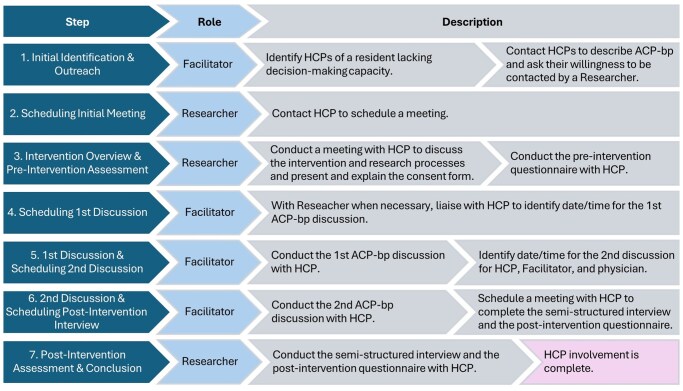
Intervention and research processes flow chart. ACP-bp = advance care planning-by proxy; HCP = health care proxy.

RACF recruitment was conducted through an advertisement about the study, which was sent to RACFs via the federation that unites them. Interested RACF directors contacted the researchers and were included in the study (no refusals were made). Health professionals within the selected RACFs were selected by the directors of care as people having enough experience and being in positions in which they would be able to conduct the interventions. Health professionals were trained by a certified ACP facilitator trainer who is responsible for the state-based traditional ACP training, in collaboration with the research team, who are also certified traditional ACP facilitators. The processes for the recruitment, intervention, and research are presented in Figure 1.

It is important to note that, in Switzerland, every person has a legal health care proxy, according to a statutory hierarchy in the civil code (Clavien et al., 2023). This hierarchy was used for the identification of health care proxies. When more than one family member wanted to participate in the discussions, they all participated.

The intervention was put into place in four RACFs in one canton of Switzerland from March 2021 until October 2023. Recruitment was stopped when the target of 20 residents was reached. This target was established through recommendations in the literature about complex intervention pilot studies, consideration of the time-intensive nature of the intervention, particularly for the RACF health professional ACP-bp facilitators, recommendations from the relevant ethics committee, and the predominantly qualitative nature of the evaluation and analysis. We anticipated that in-depth interviews with proxies of 20 residents from four different RACFs, in addition to the facilitators of the intervention, would provide us with a rich description and allow us to draw meaningful conclusions about the acceptability and feasibility of the intervention across multiple RACFs.

### Data collection

*The acceptability and feasibility of the intervention:* We conducted post-intervention semi-structured interviews with ACP-bp facilitators and proxies about their experiences with the intervention (the interview guide, developed specifically for this study, is provided in the Supplementary Material). Face-to-face interviews were conducted by a research psychologist or a research sociologist, both experienced in conducting qualitative interviews, who were not known to any of the participants outside the study context. Interviews were conducted at a time and location of the participants’ choice.

*Impact on decisional conflict for proxies*: We conducted the Decisional Conflict Scale (validated in French- (Mancini et al., 2006)) at pre- and post-intervention.

*Impact on ACP-bp facilitator (RACF health professionals) self-efficacy* in providing care in accordance with wishes: Data were collected via a numerical rating scale (1–10; developed as part of this pilot; see Supplementary Material) at pre- and post-intervention.

### Analysis

Semi-structured interviews were audio-recorded and transcribed verbatim. Thematic analysis was conducted to identify semantic level themes (Braun & Clarke, 2006). The topics of interest were established deductively, and the themes in the data were identified inductively to produce a detailed description of the important aspects of the acceptability and feasibility of the intervention. After an initial reading of the data, three researchers, LJ (researching psychologist experienced in qualitative research and analysis), YR (nurse), and EI (nurse) established an initial coding framework. One third of the data was coded in parallel, and discrepancies were discussed and resolved. Codes were compared and grouped together to develop themes. The remainder of the data was coded by LJ, according to the framework, using MAXQDA. Results of this thematic analysis are presented here, and representative verbatim quotes are presented in Tables 2 and 3 and discussed in the text.

Quantitative data were analyzed using paired samples t-tests to compare health care proxy pre-and post-intervention decisional conflict scores. Due to facilitator attrition, a lack of post-intervention facilitator self-efficacy data was collected, so statistical comparison was not possible for this outcome.

## Results

### Participants

The characteristics of the health care proxies, the residents whom the health care proxies represent, and the health professionals who facilitated the ACP-bp discussions (ACP-bp facilitators) are presented in Table 1. In ACP-bp discussions for 10 residents, two family members participated. In seven of these 10 cases, one of the two family members did not wish to participate in the acceptability and feasibility evaluation, citing a lack of time or willingness to participate in research (only proxies participating in the evaluation are presented in Table 1). For two residents, the ACP-bp facilitator and proxies decided that it was appropriate to ask the residents to participate, and the two residents accepted participation in the first discussion about their life history. Following the first discussion for these two residents, the facilitator and proxies decided that participation in the second discussion, about medical treatments, was not in the residents’ best interests. For the other 18 residents, the facilitators and proxies decided that it was not appropriate to ask the residents to participate.

**Table 1:** Health care proxy, RACF resident, and ACP-bp facilitator demographic characteristics.

Characteristics	Mean	*SD*	Range	*N* (%)
***RACF resident characteristics (n = 20)***				
**Age in years**	79.67	11.3	56–97	
**Gender**				
Female				17 (85.0)
Male				3 (15.0)
**Time in RACF in years**	2.56	1.59	0.1–10.1	
***Health care proxy characteristics (n = 21)***				
**Age**	55.7	15.6	26–76	
**Gender**				
Female				15 (71.4)
Male				6 (28.6)
**Relation to resident**				
Daughter				10 (47.6)
Son				5 (23.8)
Sister				3 (14.3)
Husband				1 (0.5)
Niece				1 (0.5)
Friend				1 (0.5)
Wife				0
***ACP-bp intervention facilitator characteristics (pre-intervention)***				
**Age in years**	46.58	13.78		
**Gender**				
Female				12 (75.0)
Male				4 (25.0)

*Note.* ACP-bp = Advance care planning-by proxy; RACF = residential aged care facility.

### Health care proxy decisional conflict

Health care proxy decisional conflict, as measured pre and post intervention by the Decisional Conflict Scale, decreased moderately (pre-intervention mean [*M*] = 57.1, *SD* = 20.25, post-intervention *M* = 44.35, *SD* = 16.21) but significantly *t*(18) = 2.2, *p* < .05 (two-tailed).

### Facilitator self-efficacy

Sixteen facilitators across four RACF were trained to conduct the ACP-bp intervention. However, only eight conducted at least one ACP-bp intervention, six of whom conducted one intervention each, and the remaining two facilitators conducted 14 interventions between them. Reasons for not conducting interventions were: left the RACF (*n* = 4), moved to a different part of the RACF (*n* = 1), moved into a different role and therefore did not have time (head nurse, *n* = 1), was completing further studies so did not have time (*n* = 1), was on sick leave and could not resume ACP-bp facilitating work when returned (*n* = 1).

Fifteen facilitators completed the self-efficacy numerical rating scale (1–10) at pre-intervention. One third of facilitators reported low to moderate self-efficacy in providing care that is coherent with resident wishes. Two-thirds of the facilitators reported high levels of self-efficacy in providing care that is consistent with resident wishes (which they explained was due to their knowledge of the residents and their experience working in the RACF context), therefore producing a relatively high average self-efficacy score for all participants pre-intervention (*M* = 7.21, *SD* = 1.57). For the facilitators who completed both the pre- and the post-intervention scale (*n* = 6), mean self-efficacy increased from 5.75 (*SD* = 1.7) pre-intervention to 9.25 (*SD* = 0.28) post-intervention; however, due to limited sample size, it is not possible to comment on the statistical significance of this increase.

Themes, sub-themes, and exemplary verbatim quotes about the acceptability and feasibility of the intervention are presented in tables and discussed in the following text.

### Acceptability of the intervention

Themes related to the acceptability of the overall process, the timing of the intervention, the guided discussions, and the documentation.

### ACP-bp facilitators

#### Overall reflections on the intervention

Facilitators reported that ACP-bp improved their knowledge of the residents, their lived experiences, their values, and their presumed will. They reported that after the intervention, they knew better how to respect the residents’ presumed wishes in case of medical emergency. They did, however, note that ACP-bp was difficult or impossible to conduct with families with complex dynamics or in distress.

#### Organizational factors

At an organizational level, facilitators indicated that having a formal ACP-bp intervention in place promoted taking initiative to make dedicated time to sit down and discuss the resident’s life in greater depth than they did before, and made ACP-bp a priority amongst busy schedules. Having a clear ACP-bp intervention also promoted more explicitly defined and distributed roles during the discussions, which allowed further depth of discussions.

#### Communication

Facilitators reported that a formal ACP-bp process promoted communication with health care proxies and allowed them to develop better relationships with the health care proxies and a better understanding of the functioning of the family system and the needs of residents. Facilitators reported that this communication promoted health care proxy confidence in the RACF staff. Facilitators also reported that the intervention promoted communication within the families, in situations when they may have otherwise avoided discussing the topics in depth. Furthermore, they reported that the intervention led to more open and collaborative discussions with the treating physicians, and promoted shared discussions and decision-making about what the residents would have wanted.

#### Timing

Facilitators reported that ACP-bp was a process that takes a considerable amount of time, but that the time taken provided valuable insights. Facilitators noted that conducting ACP-bp was much easier when they already had time to get to know the resident, but that they also needed to have the information, which they gained from ACP-bp very early in the residents’ stay, and that ACP-bp was a positive experience when it was conducted outside of emergency situations (requiring urgent decisions).

### Discussion guide

Facilitators reported discussion guides as helpful in providing them with new ways to broach a “delicate” subject (orders for medical emergency, and end-of-life situations and care). They reported that having a protocol to follow gave them permission to broach such a sensitive topic (which they had rarely done before). They also reported that having a structured and systematic guide led them to broach new topics. Although some reported following the discussion guide for the first discussions, others reported needing a more condensed format with prompts of the most important topics, and others reported that they used the guide more flexibly and adapted it to their personal style.

### Documentation

Facilitators also reported that the documents used as part of the intervention provided them with clear and understandable limits for care and instructions in case of emergency. They also appreciated the succinct format, which also allowed them to document sufficient detail and reported the importance of having a document that was standardized and made accessible to other personnel. Facilitators reported that documenting a synthesis of the resident’s values took more time and was more complex than initially expected. In addition, given the Swiss health insurance system, they indicated that space for documenting the resident’s insurance information would be a useful addition.

### Health care proxies

**Figure 2:**
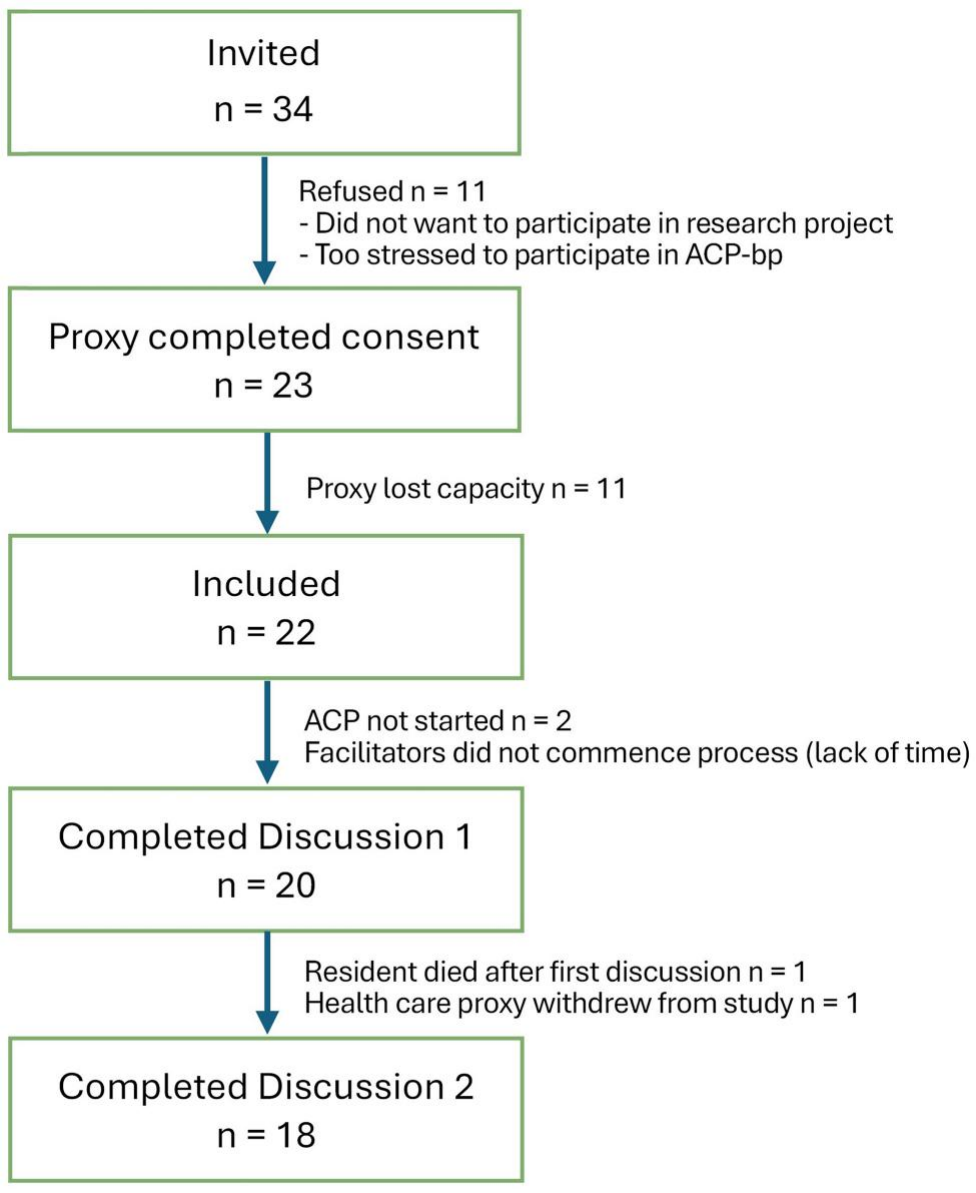
Participant recruitment flow chart. ACP-bp = advance care planning-by proxy.

Health care proxy acceptance rates could not be established as the facilitators of the intervention identified health care proxies one-by-one, based on the residents whom they believed would most benefit from the intervention and the health care proxies whom they believed would be receptive to participation, and invited them to participate individually. They did not record all potential participants; however, they did report several refusals from proxies that they contacted. These refusals were health care proxies who did not want to participate as (a) they did not want to be involved in a research project (due to previous negative experiences with research), (b) they did not want to discuss orders for emergency situations, (c) they did not feel close enough to the resident to make such decisions, and (d) they were overwhelmed by the resident’s situation and did not want to discuss emergency situations. The ACP-bp facilitators also disclosed reasons for not approaching some health care proxies for ACP-bp participation. These included having an already fragile relationship with the family, a perception that the proxy is having difficulty accepting the resident’s situation, and being unsure about whether the proxy would be competent to participate in ACP-bp (due to the proxy’s own health or cognitive state). A flow chart of health care proxy participation is provided in Figure 2, and themes relating to health care proxy acceptability from semi-structured interviews are presented in Tables 2 and 3.

**Table 2:** Themes, sub-themes, and exemplary verbatims relating to intervention acceptability from health ACP-bp facilitator interviews.

Sub-theme	Exemplary verbatim
***Theme: Overall process***	
**Knowledge of residents’ lives and presumed will**	“All of the avenues that have been brought, about the person’s values, their character, their life story, how they’ve lived their life, that gives us avenues to […] uncover the person’s own will” (Fac1)
**Difficulties with complex family dynamics**	“If there is not a realisation and acceptation, we can’t progress with the advanced directive […] we are caring for the HCP, and they aren’t the ones we are supposed to be caring for” (Fac1)
***Organizational***	
Formal ACP-bp made process a priority	“We know it’s something formalised, then at a certain time we have to take the time to do it, whatever is going on” (Fac13)
Promoted dedicated time to meet with families	“It allowed contact that we don’t always have face to face, because often we call, and we don’t have the people in front of us, we don’t live their emotions, they don’t live our emotions here and I think that it also allows us to put a face to the names” (Fac1)
Distribution of ACP-bp roles	“The doctor was more involved, because we were really in something individualised, we went further than otherwise” (Fac13)
***Communication***	
Promoted communication with HCPs	“I was surprised at how easily they (proxies) accepted it […] you need to be […] in the same channel in fact to communicate, and when we are in the same communication channel, it’s easy in fact, it’s natural” (Fac2) “The denial was present until she was with the physician, with the nurse, people who tell her concretely and help her to make a decision” (Fac1)
Promoted HCP confidence	“There is an understanding … a better understanding with families… a climate of confidence was established” (Fac2)
Promoted communication within families	“it also obliged them [within the family] to talk about things” (Fac4)
Promoted communication with physician	“It was good to have the discussion with the physician, as if not we wouldn’t have come to an understanding” (Fac3)
Promoted reflection and shared decision making	“To get everyone together for the resident, is a good thing” (Fac6) “To think together about what the person would have decided, and, in some ways, it allows us to share this decision, it doesn’t uniquely rest on one person, so it lightens the load a little” (Fac13)
***Timing***	
**Needed adequate time to know residents before doing ACP-bp**	“I know Mr so well, it’s much easier” (Fac3)
**Need to have information quickly**	“It’s essential to have information from the families very quickly in order to put things into place quickly […] it’s a tool that’s useful to put in place very quickly” (Fac2)
***Theme: Discussion guide***	
**Increased confidence and legitimacy**	“It really gave us avenues for approaching it better. Before we tried to pose the questions but it was… it’s much deeper this way” (Fac4) “I felt much more at ease […] it gave me avenues for asking the right question a the right time” (Fac1)
**Structured and systematic**	“There are questions that I wouldn’t have thought about, and, finally, it brings them together in a way that’s more structured” (Fac9)
**Flexibility in discussion guide**	“In communication, once we have an understanding of the objective, and then we can stray from the model […] to have the keywords in front of me helped me […] once we have more confidence we can add our personal touch” (Fac13)
***Theme: Documentation***	
**Provides clear limits and directions**	“The schema helps us a lot, to go over it” (Fac11) “To have a document that standardised, the same for everyone, useable, readable, in the file, clear, it really suits me” (Fac13)
**Accessible within RACF**	“We transmit the information that was collected, which allows the colleagues to also discover certain aspects of and other things that they didn’t necessarily know or they hadn’t had the occasion to go into depth with the resident” (Fac1)
**Complexity of synthesizing values**	“It’s quite complex, it occupied me for a couple of hours in the office *(laughs)*” (Fac2)
**Opportunity to formally discuss the resident**	“It allows them to see the human in the patient, it’s not ‘Madame in room 24,’ there is a story, so it’s a good process […] to know the person, where she comes from” (HCP2) “To see that someone takes the time to listen to me so I can explain what my mum is like. Because I feel like I’m putting a little gem in someone’s hands. And I’d really like to be able to feel more at peace” (HCP18) “It’s not an easy subject to broach […] I thought it was important to be able to explain what my mum was like … I think it helps them with her treatment too […] I think it was important and I think they understood” (HCP8)
**Importance of an anticipatory process**	“I find the questions completely normal and even you should be asking them […] Sometimes people don’t say anything and then all of a sudden they are demanding things and they are not in a position to judge things correctly” (HCP2) (without ACP-bp and documentation) “you come across someone in their private life, they’re there, between two things […] And then you make a quick decision. First of all, it’s not the right time, because with cell phones we can reach people at any time, anywhere, when we don’t know if they’re capable of making an important decision. And besides, verbal communication isn’t worth anything; a phone call isn’t worth anything.” (HCP18)
**Promoted reflection about medical outcomes and presumed will**	“It also makes you think about the circumstances in which you would still want medical intervention, perhaps hospitalization, if you know that your mother can recover. If her quality of life would be significantly impaired, then perhaps you would be more inclined not to seek medical intervention.” (HCP27)
**Finding common ground within families**	“Finally, she (sister) had the same opinion as me […] so finally we found an agreement” (HCP3)
**Providing agency for HCPs**	“It feels like we’re still part of our mum’s life and trying to be there for her. I see it more as a journey of love for our mum. Yeah, it’s definitely challenging, but at the same time we know we’re facing this situation. So talking about it, being able to share our thoughts, I find it quite reassuring and it helps us manage our emotions better” (HCP27)
**Emotion-laden process**	“I found it a little difficult emotionally, but I think it was quite useful for everyone” (HCP1)
**Felt listened to**	“Now I think that they know exactly what we’d like, how to react with her” (HCP6) “It’s a bit like therapy to be able to talk about things, express yourself, etc. So that’s positive” (HCP19)
***Communication***	
**Promoted open communication**	“I’ve had quite a bit of communication with the carers, the head nurse” (HCP28)
**Promoted foundations for collaboration**	“It also provides a positive foundation for support and collaboration” (HCP27)
***Timing***	
**Start ACP-bp at admission**	“I actually thought that this should have been done much earlier, right at admission” (HCP20) “Maybe before the person is admitted, it would be good to come here and have a discussion with the head nurse of the unit, to present the person (resident) […] for the personnel, it would allow them to know how to approach the person as well… ” (HCP28)

*Note.* ACP-bp = advance care planning-by proxy; Fac = ACP-bp facilitator; HCP = health care proxy; RACF = residential aged care facility.

**Table 3:** Themes, sub-themes, and exemplary verbatims relating to intervention acceptability from HCP interviews.

Sub-theme	Exemplary verbatim
***Theme: Discussion***	
**Guided discussion kept on track**	“It wasn’t just a casual discussion. There was also a protocol to the discussion, so it’s true that it perhaps brought us back to a certain medical approach. But I found that there was also quite a lot of freedom to speak, which was beneficial.” (HCP27)
**Importance of time between the two discussions**	“Discuss it […] And then think about it again and consult with the family. I think, yeah, that’s good” (HCP9)
**Promoted reflection about resident’s life**	“I found it important to be able to explain how my mother was to the (RACF)” (HCP4) “you have to think about why you’re telling the story in the first place. And then what you’re telling and why. Because then there’s your own perception of things […] you have to choose what you’re going to say. And what’s important for her here in that sense. And not just for us” (HCP19)
**Bringing together “before” and “RACF” life**	“… I learned things. For example, things about my father that I didn’t know. I think he talks differently to someone outside the family than he does to the family” (HCP32) “It helps you understand their behaviour a little better and say, ‘Ah, they react like that because that’s how they were before their illness.’ And I thought that was great. Because it’s true that if you don’t take the time to do that, you might end up with a slightly distorted image of the person.” (HCP25)
**Need for signposting during the discussions**	“During the second conversation, we would have liked to know exactly what the terms were so that we could discuss them just the two of us beforehand […] or at the beginning of the interview, starting the interview by saying, ‘We’re going to talk about this, that…’ and then we could leave for a moment and come back or something like that … I think we would have been more comfortable” (HCP8)
**Privileged time with physician**	(Int: Is there anything you particularly appreciated?) “that she (doctor) explained a bit about what happens here in relation to her health state” (HCP28) “I’m happy today about the discussion with the doctor […] And I feel relieved because it’s not always easy for me […] But this time, I was really able to say everything I was thinking. And I feel like she was very receptive.” (HCP18)
**Developed trust in physician**	“In any case, what’s also good, I think, is that it builds a relationship of trust. So already there, that means we can accept a diagnosis or advice from the doctor” (HCP27)
**Increased decision-making confidence**	“The physician was there […] and she went the same way as me when I said that she wouldn’t cope with a half a day in hospital, the physician completely agreed with me, so that convinced me that I’m making the right decisions” (HCP6)
***Theme: Documentation***	
**Reassured that decisions were documented**	“if something happens and you aren’t there, and the medical team says ‘let’s go here, let’s go there,’ it’s important, what’s wanted is noted […] we have a clear discussion. It’s correct” (HCP2)
**Clear and efficient**	“It was quite quick and efficient, I think, yes.” (HCP32)
**Promotes information transmission**	“It’s good because at least everyone will know what there is [decisions that have been made]” (HCP17)
**Provides clear examples**	“we really need to be precise (with medical orders) and explain them, so I find it super that you have been precise” (HCP5)
**Increased awareness of emergency treatments**	“There were things in the advanced directives that I learnt that I wouldn’t have imagined” (HCP5)
**Decision options are complex and abstract**	“It remains very abstract for us who are not doctors because we have to imagine everything that could happen to him. So in this case we would do this, in that case we would do that, and we don’t know. It’s huge (responsibility)” (HCP7) “It’s not intuitive for someone who isn’t in the medical field. There are lots of medical terms” (HCP19)
**Importance of physician presence for completing document**	“The doctor’s presence is important at that moment, I think” (HCP13)
**Difficulty making make concrete decisions**	“It’s a bit ambiguous, but maybe it was too precise, but it’s difficult to understand with simple examples” (HCP1) “… actually, that’s the problem: we’re being asked to do something very general, and there are so many possibilities, so many little details, so many little possibilities that… and we didn’t want him to just write ‘fracture,’ but more ‘when she’s in pain,’ for example.” (HCP7)
**Difficulty making decisions on someone else’s behalf**	“Of course, when it comes to advance directives and all that, it’s a little more complicated. It’s not that we want to decide for the other person.” (HCP23) “Well, if I’m wrong and I didn’t make the right decision, it’s true that at that moment, it stressed me out a little. Afterwards, it was fine, but there you go…” (HCP25)
**Need longer reflection time**	“It’s impossible to answer that live, like, you explain it to us, so here you go… It’s not possible. It’s something people have to take with them. Look at in a relaxed state of mind, look at it several times, ask yourself questions. Talk to those around you.” (HCP19)
**Fear of not being contacted in emergency**	“I’d still like them to call me, because there are so many possibilities that I find it difficult to know what to do” (HCP4)
**Fear of change in treatment attitude following ACP-bp documentation**	“I just want to be sure that it’s only in an emergency that they do what we’ve decided, and I don’t want what we’ve discussed to become the rule” (HCP8)
**Longitudinal process**	“something could happen, and they don’t manage to contact us, so we need something, and they also stressed that if things change in a couple of weeks, we can change it” (HCP3)

*Note.* ACP-bp = advance care planning-by proxy; Fac = ACP-bp facilitator, HCP = health care proxy; RACF = residential aged care facility.

### Overall process

Participating health care proxies reported appreciating the opportunity to discuss the residents’ lives, values, health care experiences, and future care, despite several reporting that making decisions on behalf of their loved ones was confronting and “not easy.” They recognized the importance of having anticipatory discussions when they have the time to reflect in calm conditions. They reported that the discussions promoted personal reflection about the residents’ lives and values and about their own values and wishes. Some also reported that the intervention promoted discussion within families to find a common ground for the residents’ sake. Proxies reported ACP-bp as giving them agency and a place in the residents’ care and life at the RACF. Almost all proxies noted the emotionally laden nature of the discussions, but they reported that they appreciated feeling listened to and participating in a process that reunited the people who care most closely for the resident so that all parties are on the same page and have the most important information.

#### Communication

Proxies noted that the ACP-bp process furthered a more open communication with RACF staff, and that having had the discussions has allowed the development of good foundations for future collaboration between the proxies and the RACF.

#### Timing

In terms of timing, health care proxies indicated that such discussions should be conducted as soon as possible after admission to the RACF as they felt that these discussions gave RACF staff the information they need to better know and look after residents. On the other hand, they reported that they appreciated the information about the resident’s daily life, and recognized that this takes time to establish, thus necessitating a longitudinal approach and regular ACP-bp meetings.

### Discussions

Health care proxies reported appreciating the structured nature of the discussions as it kept the conversation on track and valued the flexibility and freedom to speak. They noted the importance of having time between the two discussions as this allowed them to contact other members of the family and reflect on the first discussion before making anticipatory decisions. They also appreciated the guided discussion about the residents’ life as it helped them to reflect on the residents’ values and their own values and potential differences. Proxies were grateful for the input of the facilitators and physicians in drawing links to the residents’ life before the RACF and in the RACF and appreciated the opportunity to meet and talk with the physician in an anticipatory context rather than when problems arise. They reported that these discussions gave them more confidence and trust in the physician. Some proxies reported that they would have appreciated more signposting during the discussions, so that they could have better prepared themselves, and so that they felt more confident, especially in making anticipatory decisions. This was more pronounced when proxies reported feeling that their attitudes to care may have differed from those of the physician.

### Documentation

Health care proxies reported that they were reassured that such decisions were documented clearly, and reported the documents as being clear and efficient, and promoting information transmission. The majority appreciated the clear examples of types of situations that may require decision-making and discussed improving their awareness of such treatment options. Some proxies indicated that the treatment options were complex and abstract, thus reiterating the importance of the physician being present for medical decision-making and documentation.

Some proxies said that making concrete decisions and documenting them was difficult due to the ambiguity of the situations, and they desired space to give more detail for specific situations. They also noted the difficulty of making decisions on behalf of another person, and of documenting such decisions. Some reported needing more time to reflect before documenting such decisions.

A minority expressed concerns that by having documented decisions for emergency situations, they may not be called and informed of medical treatments in such an event, seeking reassurance that they would still be consulted. Some also reported hesitancy in documenting minimalist treatment attitudes for fear of this medical decision impacting their general care at the RACF. The possibility of changing and re-discussing the documented decisions provided reassurance for these fears.

### Feasibility of the intervention

Themes, sub-themes, and exemplary verbatim quotes relating to the feasibility of the ACP-bp intervention and suggestions for improvements are presented in Table 3, and discussed in the text.

### Factors threatening feasibility

#### Time

Both proxies and facilitators noted that the ACP-bp was time-consuming, which led to facilitators reporting a lack of dedicated time to complete ACP-bp and difficulties as they were also expected to be clinically present while doing ACP-bp. Facilitators reported the need to anticipate and block time specifically for ACP-bp. Proxies reported that some discussions were rushed, and opportunities to discuss other issues or go into more depth were limited due to a lack of time. Facilitators also reported difficulty getting in contact with proxies who have many competing demands for their time. Both facilitators and health care proxies alike indicated that the fact that there was a person dedicated to the organization of the ACP-bp (the project researcher) facilitated the intervention as it reduced the amount of work for the facilitators and provided a person easily available in case of questions. The support and reassurance of an outside person also gave facilitators confidence to pursue ACP-bp. Facilitators reported not knowing whether the ACP-bp discussions would continue to be conducted in a formal way once the pilot study ended, as this role would need to be taken up by someone whose time is already limited. In relation to the conduct of the intervention, some facilitators also discussed adapting the conduct of the intervention and conducting the two discussions at the same time to save time and facilitate organization.

#### ACP-bp champion

The facilitators who completed ACP-bp interventions discussed their roles as “champions” who promoted this process. They discussed seeing the importance of ACP-bp and feeling that if they did not promote it, it would not be done. They also discussed becoming a reference person for ACP-bp, which meant that others knew whom to contact for ACP-bp. Facilitators identified the ability to manage their own tasks and time as essential in allowing them to fit ACP-bp discussions into their schedules. The researcher also observed that having a person on each site who acted as a “champion” was most effective. Indeed, most interventions were conducted by two facilitators who championed the intervention at two sites. Several other facilitators completed only one ACP-bp intervention each. However, due to the time between training and completing the interventions (sometimes up to 1 year), this required the researcher to visit the institution to refresh their information about the process and the documents.

#### Director and physician support

ACP-bp Facilitators indicated that having an RACF director who actively encouraged ACP-bp was essential, but that even then, the clinical and practical realities of their working contexts meant that finding time to conduct ACP-bp was difficult. Physician support for ACP-bp was reported as another facilitator of the process, given the need for their presence and their relationships with the proxies. In addition, the researcher observed that having a physician who does clinical rounds on regular days of the week facilitated the organization of the second discussion. The same was true for RACFs that had a physician working nearby and being available more flexibly.

### Future directions and improvements

Suggestions for how to improve the intervention are presented in Table 4 and discussed in the following text.

**Table 4:** Feasibility and improvement suggestion themes, sub-themes, and exemplary verbatims from facilitator and health care proxy semi-structured interviews.

Sub-theme	Exemplary verbatim
***Theme: Factors threatening feasibility***	
**Time-consuming process**	“The worry is that I need to be by the residents’ side and I can’t allow myself to take time to do that [ACP], knowing, as I said, when we are on, we are the only nurse” (Fac1) The difficulty, it’s often the time factor, that’s clear […] we always have to hurry. […] In all these interviews, that’s the difficulty. It’s that we can’t be calm anymore. We have to rush to get the information out in as little time as possible (HCP18) “It’s clear that if there had been more time, perhaps we could have addressed other issues as well.” (HCP27)
**Need to plan time for ACP**	“It’s important to take ACP into account in our planning, but in the end we are able to… we are able to block out some time, but it’s about anticipating it” (Fac2)
**No dedicated time for ACP-bp**	“It hasn’t been easy, I must say, and it’s not always easy, because it’s on top of my professional duties. So, I do my best, depending on the time I have available. Sometimes I take a little longer, like this morning, when I told Dr (name) that I would process his request and draw up this advance care plan by proxy over a period of three to four weeks” (Fac15)
**Contact with proxies**	“It’s difficult to even contact them, they are difficult to get a hold of […] it’s the time, a terrible lack of time” (Fac14)
**Availability of multiple actors**	“The toughest thing was to get the families here […] they’re not always available when the doctor is” (Fac11)
**Investment of physicians**	“With the doctor, it seemed like he was stressed. He was, yeah, it felt like he just wanted it to be over as quickly as possible.” (HCP12)
**Staff turnover**	“Because of Covid it’s not easy, well, we also have lots of organisational problems […] the head nurse left” (Fac12) “So I think there seems to be a lot of turnover in the teams.” (HCP8)
**Emotional nature of ACP-bp**	“The emotional charge, it’s a weight for the loved ones, and also for oneself, as a carer, even if we put on the white coat, we are professionals, but we are still human, we all have families, we all have loved ones” (Fac14)
***Theme: Factors promoting feasibility***	
**ACP-bp recognized as important work**	“It must be something that plays a central role in our work.” (Fac15)
**Dedicated organizational resource (researcher)**	“To have someone with me, it gave me confidence, to take on that role” (Fac13)
**Adapting the intervention**	Fac15: We get straight to the point with the relative, defining the therapeutic objectives and reviewing the person’s values and life history. INT: So instead of having two discussions… Fac15: Exactly, we do a condensed version where we cover all these elements in a single interview.
**“champions” to promote ACP-bp**	“I knew I was invested in this role for (name of RACF) and I had to invest more because I was the only reference for this project” (Fac2) “I get the impression that, by default, people think, ‘Oh, that’s (name of Facilitator),’ both doctors and my colleagues, for example” (Fac15)
**Facilitator flexibility to manage workload**	“Then I would say that because of my position, I am responsible, I have a certain amount of leeway. And then, at some point, I can also decide, for example, that there are certain tasks that I can delegate. You see, to my colleagues, and then, by concentrating and freeing up time for ACP-bp. I would say that this is ultimately an advantage that I have.” (Fac15)
**Support from director**	“we said with (director) that we need to account for it in the planning, but finally we are able to” (Fac13)
**Proactive physician**	“Above all, there was Dr (name), who was a driving force in all of this, so he was a great resource” (Fac15)
***Theme: Suggestions for improving the intervention***	
**Need for more time/preparation**	“But there were lots of things to do. So maybe do one thing. So first, we’ll start with that. Maybe we’ll send it to your home or send it to you by mail. We’ll let you read it so you’re prepared” (HCP25)
**Involve referent carers**	“What about the carers, it could be important to have the carers, those who are beside the resident every day, also present, so that they know them better” (HCP17)
**Possibilities to change/follow up decisions**	“Make it easier if we want to change our decision, there isn’t… a website where we put our wishes, it’s an interview, it’s on a piece of paper, and we can’t see it. Nowadays, it’s all about the internet and things like that, but maybe this kind of thing is too personal to be on the internet, I don’t know. Maybe it would make it easier to make decisions, I don’t know if that’s feasible.” (HCP7)
**Need for more concrete examples**	“I think there could have been more explanation of comfort care, relentless treatment, but I imagine there are so many different examples that we can’t review them all” (HCP1) “It would be good to have a description of what we can do […] We could facilitate these discussions if we knew everything that was possible or not possible.” (HCP32)

*Note.* ACP = advance care planning; ACP-bp = ACP-by proxy; Fac = ACP-bp facilitator; HCP = health care proxy; RACF = residential aged care facility.

In line with previously discussed lack of time, all parties reported needing more time to be allocated to conducting ACP-bp. Proxies also suggested that the documents and discussion guides could be given in advance, to allow proxies to better prepare themselves. Some proxies also reported that, in addition to the facilitator and the physician, they would have appreciated the presence of the carers who are most often with the resident, so that they could get more information about the resident’s daily life and share their knowledge of the resident with these carers. Proxies also expressed a desire for a tool that allowed them to access the decisions, and to update them as needed. They also suggested that written support materials which explain medical terminology, give more information about end of life in RACFs, and concrete examples of treatment and care options as well as experiences of proxies in similar situations, and available support services for proxies would be useful additions to the intervention.

## Discussion and implications

The results of this study provide important insights into how ACP-bp models could work in the RACF context. The intervention led to a significant decrease in health care proxy decisional conflict as well as an increase in facilitator self-efficacy in giving care that is coherent with the residents’ wishes. These results, despite the limited sample size of a pilot study, are consistent with the only existing interventional study from Canada showing that increasing communication between family members and health professionals and discussing future care planning are beneficial and significantly reduce health care proxy decisional conflict (Brazil et al., 2018).

Furthermore, the results of our study suggest that the benefits of traditional ACP, such as improvement of communication with physicians, and reduction in decisional conflict (Malhotra et al., 2022) may also be achievable when ACP is conducted via health care proxies. As ACP-bp was reported to improve communication between RACF physicians, health professionals and health care proxies and establishes long-term goals of care based on the resident’s presumed will, this model may help to avoid reasons for preference discordance between people with severe dementia and their health care proxies, such as fears about making the “wrong” decision, and disagreement over long-term goals of care identified in previous research (Malhotra et al., 2021). Improved communication, mutual understanding, and clear plans for emergency decisions may also reduce health care proxy stress and support them in their role as substitute decision makers (Cresp et al., 2020).

Previous research has highlighted the lack of a single tool that encompasses all caregiver decision-making needs across the domains of promoting communication, family support, explaining proxy roles, recognizing proxies and their roles, reducing decision making uncertainty, and providing information about the progression of dementia (King et al., 2024). The results of this pilot lend support to the use of ACP-bp models to be able to provide support to each of these domains. Our findings that facilitators felt ACP-bp improved their understanding of residents and their families, and that health care proxies felt that their loved ones were better understood, support the importance of these factors already noted in the literature (Konno et al., 2024).

Our results also show that the intervention was acceptable to those who participated (health care proxies and facilitators alike), but there were multiple factors that threatened the feasibility of the intervention. These factors included a lack of facilitator, physician, and health care proxy time, difficulties organizing the discussions, and RACF staff turnover. It is important to note that the two facilitators who conducted the majority of the ACP-bp discussions reported “creating” time for ACP-bp and having the flexibility in their schedules to do so. These two “champions” of the process were a social worker and a head nurse. This provides us with important insights into the positions best placed to include ACP-bp in their work. In Switzerland, it is rare to have social workers employed in RACFs; however, our findings, along with previous literature (Wang et al., 2017) lend support to the utility of social workers facilitating ACP-bp.

If models of ACP-bp are to be implemented more systematically, greater awareness and readiness are needed by RACF health professionals, physicians, and health care proxies. In order to increase awareness, specific trainings are needed to explain the purpose and process of ACP-bp as well as each actor’s role in this process, as suggested for traditional ACP in RACFs (Flo et al., 2016). In addition, as ACP-bp is a time-consuming intervention necessitating physicians, health care proxies, and facilitators meeting together, the time for clinicians and for the organization needs to be factored into RACF budgets (and health insurance billing structures, where relevant) and organizational structures. Time barriers have been reported in previous reviews on ACP implementation and implementation of family conferencing in RACFs (Luckett et al., 2017). Another systematic review has identified preconditions for implementing ACP in the RACF context in the domains of knowledge and skills, willingness and ability to participate, relationships, administrative systems, and contextual factors (Gilissen et al., 2017). An international multiple-case study has also highlighted the importance of supportive relationships, and committed staff who see the intervention as valuable, in implementing ACP in RACF settings (Brazil et al., 2024). The results of this pilot further highlight the importance of relationship building, which was promoted through ACP-bp, and the importance of administrative systems and context in the feasibility of an acceptable intervention. Increasing RACF health professional self-efficacy in conducting ACP is also essential to improving feasibility (Gilissen et al., 2020). Given widespread limitations in resources, ACP-bp feasibility could be increased in the RACF setting by holding group information sessions for proxies, which could prepare them for their roles as proxies, and explain ACP-bp concepts and procedures, thus engaging them in the initial stages of change and preparing them for ACP-bp. This could promote more efficient ACP-bp coordination and organization processes and save time during the actual discussions.

### Limitations

There are several limitations to this pilot study. The residents for whom proxies completed ACP-bps in this study were representative of the age demographics of the average age of RACF residents in the state in which the pilot was conducted. However, it is not possible to comment on the representativeness of the health care proxies as there is no data available about the health care proxies for comparison. In both the health care proxies and facilitator groups, the majority of participants were female, which is reflective of the gender differences in both formal and informal caregiving role distribution, and also in traditional ACP participation. This is likely to have arisen due to a selection bias stemming from cultural and familial norms, and health professional- proxy interactions, and highlights the need to be attentive to encouraging and inviting male participation in formal and informal caring roles.

Another major limitation relates to the screening and inclusion of potential health care proxy participants. ACP-bp facilitators anecdotally discussed approaching families with whom they have had positive contact, or whom they thought would be open to participating. Initially, it was envisaged that all eligible families would be contacted; however, given the naturalistic context of the intervention, the lack of national and institutional directives for ACP in RACFs, and a general reluctance to initiate ACP, we did not have control or power to influence the selection of proxies who were included, therefore opening up a greater risk of selection bias. Future research should seek to establish an institutional directive for ACP-bp within which to situate an intervention study with a more robust inclusion process. In addition, the RACFs that volunteered to participate were highly interested in the topic and invested in improving their care planning practices. Therefore, the limited feasibility of the model is likely to be exacerbated in RACFs with fewer resources and less motivation, or with proxies who are coping less well. This model was implemented in a French-speaking region of Switzerland, and the results therefore need to be considered through the lens of the Swiss insurance-based health system and the cultural and linguistic characteristics of this region.

The process of completing lengthy consent forms turned some health care proxies off participating in the research, even though they said they would have been happy to participate in the intervention. This was mainly due to previous negative experiences with research projects. Researchers must thus take extra care to ensure that research participation is a positive experience for health care proxies and people with dementia and that they maintain an open and coherent dialogue with participants throughout the experience.

### Future directions

Despite the high level of acceptability and many benefits being identified, several improvements should be made to the ACP-bp model. What became obvious is the need for specific tools to prepare proxies for ACP-bp discussions and decisions. Such resources should provide proxies with information about the treatments available in RACFs, medical terminology, the ACP-bp process, the proxy’s role in it, and any available supports for proxies. A review of such supports in the Australian context can give insights into the development of such resources (Tran et al., 2021).

The high level of acceptability combined with feasibility difficulties highlighted in this study indicate the need to consider which models are the most appropriate for implementing ACP-bp in the RACF context. Models which may be considered include (a) a “champion” in the RACF who trains other health professionals and takes a proactive role in organizing ACP-bp discussions, (b) a “pool” of external facilitators who conduct ACP-bp discussions in a range of RACFs, and (c) having all registered nurses or other staff (e.g., psychologists and social workers) in the RACFs trained so that they can conduct ACP-bp interchangeably. These models should be tested for feasibility.

Future research in this area should focus on the evaluation of larger-scale implementation of such an intervention. Ideally, a cluster randomized trial should be conducted over a medium-term timeframe, which can establish the effect of ACP-bp on outcome measures such as care that is coherent with patient wishes. In addition, long-term studies could be conducted to establish proxy and health professional experiences with the usefulness of ACP-bp processes and documents when situations arise in which decisions need to be made or when documents need to be updated. Further insights into the successful implementation, in order to overcome the aforementioned difficulties, can be gained from implementation sciences.

ACP-bp has the potential to be a feasible approach that is accepted by health professionals, physicians, and health care proxies in RACF. Multiple benefits regarding the care of the residents became obvious in this pilot study. Future studies have to tackle contextual barriers that threaten the feasibility of ACP-bp and prove its effectiveness on a larger scale.

## Supplementary Material

igag033_Supplementary_Data

## Data Availability

The datasets generated or analyzed during the current study are not publicly available as these data are primarily qualitative and publishing them may provide enough information to identify the participants. Selected extracts have been translated and anonymized for publication here. Data will be made available upon reasonable request from the corresponding author for the purposes of verification.

## References

[igag033-B1] Baumann S. M. , Kruse N. J., Kliem P. S., Amacher S. A., Hunziker S., Dittrich T. D., Renetseder F., Grzonka P., Sutter R. (2023). Translation of patients’ advance directives in intensive care units: Are we there yet? Journal of Intensive Care, 11, 53. 10.1186/s40560-023-00705-z 37968692 PMC10648602

[igag033-B2] Blake M. , Doray O. N., Sinclair C. (2017). Advance care planning for people with dementia in Western Australia: An examination of the fit between the law and practice. Psychiatry, Psychology and Law, 25, 197–218. 10.1080/13218719.2017.1351904 PMC6818247 31984016

[igag033-B3] Bosisio F. , Jox R. J., Jones L., Rubli Truchard E. (2018). Planning ahead with dementia: What role can advance care planning play? A review on opportunities and challenges. Swiss Medical Weekly, 148, 1–9. 10.4414/smw.2018.14706 30594990

[igag033-B4] Bosisio F. , Sterie A.-C., Rubli Truchard E., Jox R. J. (2021). Implementing advance care planning in early dementia care: Results and insights from a pilot interventional trial. BMC Geriatrics, 21, 1–11. 10.4414/smw.2018.14706 34666711 PMC8524211

[igag033-B5] Braun V. , Clarke V. (2006). Using thematic analysis in psychology. Qualitative Research in Psychology, 3, 77–101. 10.1191/1478088706qp063oa

[igag033-B6] Brazil K. , Carter G., Cardwell C., Clarke M., Hudson P., Froggatt K., McLaughlin D., Passmore P., Kernohan W. G. (2018). Effectiveness of advance care planning with family carers in dementia nursing homes: A paired cluster randomized controlled trial. Palliative Medicine, 32, 603–612. 10.1177/0269216317722413 28786323

[igag033-B7] Brazil K. , Walshe C., Doherty J., Harding A. J., Preston N., Bavelaar L., Cornally N., Di Giulio P., Gonella S., Hartigan I. (2024). Implementation of an advance care planning intervention in nursing homes: An international multiple case study. The Gerontologist, 64, gnae007. 10.1093/geront/gnae007 PMC11102006 38349015

[igag033-B8] Chan F. , Woo J. (2025). Factors affecting the initiation and consideration of advance care planning (ACP) with surrogates of older adults with advanced dementia: A systematic review. Advances in Geriatric Medicine and Research, 7, 1–21. 10.20900/agmr20250005

[igag033-B9] Clavien C. E. , Ulrike, Jox R., Karzig I., Krones T., Loupatzatzis B., Monteverde S., Theile G. (2023). Advance care planning in Switzerland: Chances and challenges of delivering high-quality ACP in a small high-income, multilingual, federally organized country. Zeitschrift für Evidenz, Fortbildung und Qualität im Gesundheitswesen, 180, 115–120. 10.1016/j.zefq.2023.04.008 37438170

[igag033-B10] Cohen A. B. , DeMartino E. S. (2021). How should advance care planning be done when a surrogate is making decisions? Journal of the American Geriatrics Society, 69, 2103. 10.1111/jgs.17222 34002373 PMC8373712

[igag033-B11] Coors M. , Jox R. (2015). Advance care planning: Von der Patientenverfügung zur gesundheitlichen Vorausplanung. Kohlhammer Verlag. 10.17433/978-3-17-028675-7

[igag033-B12] Craig P. , Dieppe P., Macintyre S., Michie S., Nazareth I., Petticrew M. (2013). Developing and evaluating complex interventions: The new Medical Research Council guidance. International Journal of Nursing Studies, 50, 587–592. 10.1016/j.ijnurstu.2012.09.010 23159157

[igag033-B13] Cresp S. J. , Lee S. F., Moss C. (2020). Substitute decision makers’ experiences of making decisions at end of life for older persons with dementia: A systematic review and qualitative meta-synthesis. Dementia, 19, 1532–1559. 10.1177/1471301218802127 30253658

[igag033-B14] Dening K. H. , Greenish W., Jones L., Mandal U., Sampson E. L. (2012). Barriers to providing end-of-life care for people with dementia: A whole-system qualitative study. BMJ Supportive & Palliative Care, 2, 103–107. 10.1136/bmjspcare-2011-000178 24654049

[igag033-B15] Detering K. M. , Buck K., Ruseckaite R., Kelly H., Sellars M., Sinclair C., Clayton J. M., Nolte L. (2019). Prevalence and correlates of advance care directives among older Australians accessing health and residential aged care services: Multicentre audit study. BMJ Open, 9, e025255. 10.1136/bmjopen-2018-025255 PMC6340468 30647047

[igag033-B16] Detering K. M. , Hancock A. D., Reade M. C., Silvester W. (2010). The impact of advance care planning on end of life care in elderly patients: Randomised controlled trial. BMJ, 340, c1345. 10.1136/bmj.c1345 20332506 PMC2844949

[igag033-B17] Dixon J. , Karagiannidou M., Knapp M. (2018). The effectiveness of advance care planning in improving end of life outcomes for people with dementia and their carers: A systematic review and critical discussion. Journal of Pain and Symptom Management, 55, 132–150. 10.1016/j.jpainsymman.2017.04.009 28827062

[igag033-B18] Fagundes D. F. , Costa M. T., Alves B. B. d. S., Benício M. M. S., Vieira L. P., Carneiro L. S., Nascimento O. J. M., Monteiro Junior R. S. (2021). Prevalence of dementia in long-term care institutions: A meta-analysis. Jornal Brasileiro de Psiquiatria, 70, 59–67. 10.1590/0047-2085000000298

[igag033-B19] Flo E. , Husebo B., Bruusgaard P., Gjerberg E., Thoresen L., Lillemoen L., Pedersen R. (2016). A review of the implementation and research strategies of advance care planning in nursing homes. BMC Geriatrics, 16, 24. 10.1186/s12877-016-0179-4 26797091 PMC4722739

[igag033-B20] Garvelink M. M. , Boland L., Klein K., Nguyen D. V., Menear M., Bekker H. L., Eden K. B., LeBlanc A., O’Connor A. M., Stacey D. (2019). Decisional conflict scale findings among patients and surrogates making health decisions: Part II of an anniversary review. Medical Decision Making, 39, 316–327. 10.1177/0272989X19851346 31142205

[igag033-B21] Gilissen J. , Pivodic L., Smets T., Gastmans C., Vander Stichele R., Deliens L., Van den Block L. (2017). Preconditions for successful advance care planning in nursing homes: A systematic review. International Journal of Nursing Studies, 66, 47–59. 10.1016/j.ijnurstu.2016.12.003 27987411

[igag033-B22] Gilissen J. , Pivodic L., Wendrich-van Dael A., Cools W., Vander Stichele R., Van den Block L., Deliens L., Gastmans C. (2020). Nurses’ self-efficacy, rather than their knowledge, is associated with their engagement in advance care planning in nursing homes: A survey study. Palliative Medicine, 34, 917–924. 10.1177/0269216320916158 32383636

[igag033-B23] Goldstein N. E. , Mather H. (2018). The complexities of advance care planning in individuals with advanced dementia. JAMA Internal Medicine, 178, 969–970. 10.1001/jamainternmed.2018.1490 29868779

[igag033-B24] Gozalo P. , Teno J. M., Mitchell S. L., Skinner J., Bynum J., Tyler D., Mor V. (2011). End-of-life transitions among nursing home residents with cognitive issues. New England Journal of Medicine, 365, 1212–1221. 10.1056/NEJMsa1100347 21991894 PMC3236369

[igag033-B25] Hamann J. , Bronner K., Margull J., Mendel R., Diehl‐Schmid J., Bühner M., Klein R., Schneider A., Kurz A., Perneczky R. (2011). Patient participation in medical and social decisions in Alzheimer’s disease. Journal of the American Geriatrics Society, 59, 2045–2052. 10.1111/j.1532-5415.2011.03661.x 22092150

[igag033-B26] Hedinger D. , Braun J., Zellweger U., Kaplan V., Bopp M., Group S. N. C. S. (2014). Moving to and dying in a nursing home depends not only on health–An analysis of socio-demographic determinants of place of death in Switzerland. PLoS One, 9, e113236. 10.1371/journal.pone.0113236 25409344 PMC4237376

[igag033-B27] in der Schmitten J. , Jox R. J., Pentzek M., Marckmann G. (2021). Advance care planning by proxy in German nursing homes: Descriptive analysis and policy implications. Journal of the American Geriatrics Society, 69, 2122–2131. 10.1111/jgs.17147 33951187

[igag033-B28] Jones L. , Rhyner F., Rutz Voumard R., Figari Aguilar F., Rubli Truchard E., Jox R. J. (2024). “What Is the Most Important to Them?” Swiss health care proxies, nurses, and physicians discuss planning practices for aged care residents who no longer have medical decision-making capacity. Gerontology, 70, 173–183. 10.1159/000535455 38008064 PMC10866175

[igag033-B29] Jones L. , Rutz Voumard R., Rhyner F., Figari Aguilar F., Rubli Truchard E., Jox R. J. (2025). Proxy, nurse, and physician needs regarding advance care planning by proxy for aged care residents lacking decision making capacity: An exploratory study. BMC geriatrics, 25, 728. 10.1186/s12877-025-06354-1 41013250 PMC12465167

[igag033-B30] Jox R. (2016). Lost decisional capacity- lost chance of advance care planning?. Bioethica Forum, 9, 109-110. 10.24894/BF.2016.09028

[igag033-B31] Jox R. J. (2019, 13-16 March 2019). Advance care planning by proxy: An ethico-legal analysis (O74). Advance Care Planning International (ACP-I).

[igag033-B32] King S. , Fernandes B., Jayme T. S., Boryski G., Gaetano D., Premji Z., Venturato L., Santana M. J., Simon J., Holroyd‐Leduc J. (2024). A scoping review of decision‐making tools to support substitute decision‐makers for adults with impaired capacity. Journal of the American Geriatrics Society, 72, 2219–2231. 10.1111/jgs.18812 38400764

[igag033-B33] Konno R. , Inoue K., Matsushita Y., Hashimoto K., Wiechula R., To T., Schultz T. J. (2024). Barriers to advance care planning in older adults with dementia, their families and healthcare professionals: An umbrella review of qualitative evidence. Research on Aging, 46, 339–358. 10.1177/01640275241227909 38242164

[igag033-B34] Lepping P. , Stanly T., Turner J. (2015). Systematic review on the prevalence of lack of capacity in medical and psychiatric settings. Clinical Medicine, 15, 337–343. 10.7861/clinmedicine.15-4-337 26407382 PMC4952795

[igag033-B35] Luckett T. , Chenoweth L., Phillips J., Brooks D., Cook J., Mitchell G., Pond D., Davidson P. M., Beattie E., Luscombe G. (2017). A facilitated approach to family case conferencing for people with advanced dementia living in nursing homes: Perceptions of palliative care planning coordinators and other health professionals in the IDEAL study. International Psychogeriatrics, 29, 1713–1722. 10.1017/S1041610217000977 28651659

[igag033-B36] Malhotra C. , Mohamad H., Østbye T., Pollak K. I., Balasundaram B., Malhotra R., Tong K.-M., Hum A. Y. M., Allen J. C., Seow D. J. (2021). Discordance between dementia caregivers’ goal of care and preference for life-extending treatments. Age and Ageing, 50, 1382–1390. 10.1093/ageing/afab049 33890622

[igag033-B37] Malhotra C. , Shafiq M., Batcagan-Abueg A. P. M. (2022). What is the evidence for efficacy of advance care planning in improving patient outcomes? A systematic review of randomised controlled trials. BMJ Open, 12, e060201. 10.1136/bmjopen-2021-060201

[igag033-B38] Mancini J. , Santin G., Chabal F., Julian-Reynier C. (2006). Cross-cultural validation of the Decisional Conflict Scale in a sample of French patients. Quality of life Research, 15, 1063–1068. 10.1007/s11136-005-6003-9 16900286

[igag033-B39] McMahan R. D. , Knight S. J., Fried T. R., Sudore R. L. (2013). Advance care planning beyond advance directives: Perspectives from patients and surrogates. Journal of Pain and Symptom Management, 46, 355–365. 10.1016/j.jpainsymman.2012.09.006 23200188 PMC4111444

[igag033-B40] Meier C. , Vilpert S., Wieczorek M., Borrat-Besson C., Jox R. J., Maurer J. (2024). End-of-life health literacy, knowledge and behaviours towards advance care planning among older adults: Cross-sectional evidence from Switzerland. BMJ Public Health, 2, 1–11. 10.1136/bmjph-2023-000600 PMC11812822 40018165

[igag033-B41] Merche J. , Thonon H., Sibille F.-X., Gabriel J., Simonin E., Schoevaerdts D., Van Durme T., de Saint-Hubert M. (2025). Avoidable emergency department admissions among nursing home residents–insights from a retrospective study. European Geriatric Medicine, 17, 1–15. 10.1007/s41999-025-01264-2 40610839 PMC12946282

[igag033-B42] Morris P. , McCloskey R., Keeping-Burke L., Manley A. (2021). Nurses’ provisions for self-determination in residents with cognitive impairment who live in a residential aged care facility: A scoping review. JBI Evidence Synthesis, 19, 1583–1621. 10.11124/JBIES-20-00291 36521065

[igag033-B43] Parker D. (2010). Palliative care in residential aged care facilities. Progress in Palliative Care, 18, 352–357. 10.1179/1743291X10Y.0000000005

[igag033-B44] Poole M. , Bamford C., McLellan E., Lee R. P., Exley C., Hughes J. C., Harrison-Dening K., Robinson L. (2018). End-of-life care: A qualitative study comparing the views of people with dementia and family carers. Palliative Medicine, 32, 631–642. 10.1177/0269216317736033 29020864

[igag033-B45] Rietjens J. A. , Sudore R. L., Connolly M., van Delden J. J., Drickamer M. A., Droger M., van der Heide A., Heyland D. K., Houttekier D., Janssen D. J. J. T. L. O. (2017). Definition and recommendations for advance care planning: An international consensus supported by the European Association for Palliative Care. 18, e543–e551. 10.1016/S1470-2045(17)30582-X 28884703

[igag033-B46] Shalowitz D. I. , Garrett-Mayer E., Wendler D. (2006). The accuracy of surrogate decision makers: A systematic review. Archives of Internal Medicine, 166, 493–497. 10.1001/archinte.166.5.493 16534034

[igag033-B47] Tewari R. , Piovezan R. D., Jadczak A. D., Visvanathan R. (2025). Underdiagnosis of dementia in residents of residential aged care services: A scoping review. Australasian Journal on Aging, 44, e70030. 10.1111/ajag.70030 PMC12012595 40259830

[igag033-B48] Tran J. , Sellars M., Nolte L., White B. P., Sinclair C., Fetherstonhaugh D., Detering K. (2021). Systematic review and content analysis of Australian health care substitute decision making online resources. Australian Health Review, 45, 317–327. 10.1071/AH20070 33472740

[igag033-B49] Volicer L. , Cantor M. D., Derse A. R., Edwards D. M., Prudhomme A. M., Gregory D. C. R., Reagan J. E., Tulsky J. A., Fox E. (2002). Advance care planning by proxy for residents of long‐term care facilities who lack decision‐making capacity. Journal of the American Geriatrics Society, 50, 761–767. 10.1046/j.1532-5415.2002.50175.x 11982681

[igag033-B50] Wang C.-W. , Chan C. L., Chow A. Y. (2017). Social workers’ involvement in advance care planning: A systematic narrative review. BMC Palliative Care, 17, 5. 10.1186/s12904-017-0218-8 28693527 PMC5504662

[igag033-B51] Zhai S. , Lu Y., Liu Q., Dai C., Chen C. (2024). Factors influencing dementia patients’ participation in advance care planning: A meta-analysis. Geriatric Nursing, 60, 469–480. 10.1016/j.gerinurse.2024.10.014 39426271

