# Supplementary material for: Advance care planning by proxy for residential aged care facility residents: results of a pilot interventional study
Source: Innov Aging. 2026 Apr 7;10(6):igag033. doi: 10.1093/geroni/igag033 (PMC13161567; doi:10.1093/geroni/igag033)
Supplement: igag033_Supplementary_Data [file igag033_supplementary_data.pdf]

***Innovation in Aging* Supplementary Material: Jones et al. Advance care planning by proxy for residential aged care facility residents: results of a pilot interventional study.**

ACP-bp discussion guide
Discussion 1

Preamble

In order to ensure that your (relation) receives the best care and that any medical treatment is coherent with their wishes, we would like to find out more about their life and the things that are most important in their life. As they are no longer able to tell us what they want in terms of care and treatment, we have to rely on information provided by people close to them. It is important that, when we discuss their wishes, we think about what they would have wanted if they had been able to tell us, and <u>not think about our own wishes</u>.

Life story

To begin with, can you tell me about your (relation)'s life?

- *Can you give me a **brief overview of** their life, family and work?*
- *Can you talk me about their **character/personality**?*
- *What do they **like to do**? Is there anything you can think of that they **don't like**?*
- ***Who are the most important people** in their life?*
  (Are there any family members with whom they do not get on well?)

Values

What is the **most important thing in their life** right now? What gives their life **meaning**?

- *How did they communicate this to you? When did you discuss it? In which context?*
- *Has this changed over the course of their life? In what way?*

Have they **discussed** their personal **values** with you? With **other family members**?

- *What values did you talk about? When did they talk about them? In which context?*
- *Has this changed over the course of their life? How has it changed?*

Are these values coherent with **your own**? How are they **similar**? How are they **different**?

Do they have any **religious** or **spiritual** convictions?

- *Can you tell me more?*
  - *What impact do these convictions have on their life?*
  - *Are there any rituals that are important to them?*
- *Have these convictions changed over the course of their life?*

Previous experience of illness and the healthcare system

<u>*Facilitator: (Referring to the medical file, discuss previous medical interventions)*</u>
How did they **experience this treatment/intervention**? Do you think that if a similar problem were to arise, they would want the same type of care? *Why or why not?*

Have there been any **events** in **your family** (e.g. hospital admissions, intensive medical treatment or death) that have led to discussions about what (resident) would or would not want in a similar situation?

Have they indicated any **preference for medical care**? When was this? In which context? Do you think these preferences are still valid today? *Why/ why not?*

Previous documentation

Has (resident) ever **documented** their values/wishes for the future? In what form? Where is this document kept? Who has access to it?

- Have they ever documented their wishes regarding **future medical care**, for example, resuscitation? When did they document this/in which context? What were their wishes?
    - Do you think this document **still** reflects their wishes? *Why or why not?*

Closure of the first discussion

Thank you for this valuable information. I will summarise it and we will check it together at our next discussion on (date); it is also very important that we decide what measures to put in place for (name) one in the event of an emergency- we will discuss this at the next meeting, where the physician will also be present.

Discussion 2

(general goals of care, anticipation of health issues, anticipatory decisions for emergency treatment, documentation)

Review of the summary of values

Facilitator: describes the values identified in the first part

- Does the **summary of values** we have identified seem correct to you? Have you thought of **anything else** meanwhile? Is there anything important missing?
- Do you think we can use these values and preferences as a **basis for making anticipatory decisions** about medical treatment and care on behalf of (resident)?

General attitude towards care and treatment

In the light of the values you have identified and your knowledge of (resident), what do you believe their **general goals of care** would be ?

Is it important to (name) that **their life be prolonged**? Have they talked to you about this? Why do you think this?

- Do you think they would want treatments to **prolong their life** even if their quality of life deteriorated?
- Do you think they would want to prioritize care to keep them **comfortable in** the knowledge that such treatment may mean that their life is not being prolonged?

<u>→ note the general goals of care in the documentation</u>

Emergency orders

Now that we have established the general goals of care that (state attitude), we need to identify what types of treatment (resident) would want in an emergency situation (in case we need to make a decision very quickly and don't have time to discuss this in detail with you).

Take lead from the general attitude, use the grid in the documentation

No life-prolonging treatments (start with comfort care to develop a common understanding of what "comfort care only" means):

Comfort care→ Treatment to prolong life in RACF ? → Transfer to hospital for diagnosis and return to RACF?

Treatments designed to **prolong life in certain circumstances** (starting with comfort care):

Comfort care→ Treatment to maintain life in RACF ? → Transfer to hospital for diagnosis and return to RACF? → Treatment in hospital but no intensive care ? → Intensive care? → Mechanical ventilation?

All life-sustaining measures (start from resuscitation and work backwards):

Resuscitation? → Mechanical ventilation ? → Intensive care treatment ?

→ document emergency orders in the documentation

Other decisions

Preamble: It is now important to review (name)'s medical history and try to anticipate any situation in which a decision about their medical treatment and care may be necessary.

Current state of health

Review of medical history

Potential future health problems

Physician explains possible changes in the state of health

Care available / treatment options

Explain the medical indication (for certain pathologies, several treatment options are possible, but some may not be beneficial or medically indicated (and are therefore not proposed) for certain patients depending on their specific state of health.

In the case of your (relative), the following treatments would be medically indicated (xxxxx) for (conditions xxxx).

→ note anticipatory decisiosn for other conditions in the "other medical orders" section of the documentation

Review/check consistency with values

Are you satisfied with these decisions? Are they what **your (relative) would have wanted** in these situations?

- Are they **consistent with the values** identified during our last discussion?
- Are they in **line with the** general **goals of care**?
- Are there any aspects you're **not sure** about?

ACP-bp documentation

Advance Care Planning by Proxy (with Health Care Proxy)

Document for Advance Care Planning by proxy for internal Residential Aged Care Facility use

This document is a declaration of the presumed will of a person who, at the time of completing this document, no longer has decision making capacity and is no longer able to make decisions about their medical treatment. It is not expected that this person will regain the capacity to make their own medical decisions. This document is based on in-depth discussions between the legal health care proxy of the person who no longer has medical decision making capacity and a facilitator trained to conduct advance care planning by proxy discussions.

RESIDENT INFORMATION

Family Name ____________________ First Name(s) ____________________

Date of birth ____________________ Name of RACF ____________________

IMPORTANT MEDICAL INFORMATION

The resident has the following diagnoses:

SYNTHESIS OF VALUES

GENERAL GOALS OF CARE

□ ***Symptom management only****, no treatments intended to prolong life*

□ *Treatment to* ***prolong*** *life in* ***certain circumstances****. Define the circumstances :*

□ ***All medically indicated treatment*** *aiming to prolong life*

Comments :

MEDICAL ORDERS IN CASE OF EMERGENCY		
	**Accepted Treatment**	**Refused treatment**
	**Comfort care**	**Treatment aiming to prolong life at the RACF,** Transfer to hospital if a diagnosis is not possible at the RACF followed by a return to the RACF**,** Treatment in hospital, Intensive care treatment, mechanical ventilation, Cardio-Pulmonary Resuscitation
	**Treatment aiming to prolong life at the RACF** (eg : antibiotics), comfort care.	**Transfer to hospital if a diagnosis is not possible at the RACF followed by a return to the RACF,** Treatment in hospital, Intensive care treatment, mechanical ventilation, Cardio-Pulmonary Resuscitation
	**Transfer to hospital if a diagnosis is not possible at the RACF followed by a return to the RACF,** treatment aiming to prolong life at the RACF, comfort care.	**Treatment in hospital,** Intensive care treatment, mechanical ventilation, Cardio-Pulmonary Resuscitation
	**Treatment in hospital**, transfer to hospital if a diagnosis is not possible at the RACF, treatment aiming to prolong life at the RACF, comfort care.	**Intensive care treatment**, mechanical ventilation, Cardio-Pulmonary Resuscitation
	**Intensive care treatment**, treatment in hospital, transfer to hospital if a diagnosis is not possible at the RACF, treatment aiming to prolong life at the RACF, comfort care.	**Mechanical ventilation**, Cardio-Pulmonary Resuscitation
	**Mechanical ventilation,** intensive care treatment, treatment in hospital, transfer to hospital if a diagnosis is not possible at the RACF, treatment aiming to prolong life at the RACF, comfort care.	**Cardio-Pulmonary Resuscitation**
	**Cardio-Pulmonary Resuscitation,** mechanical ventilation, intensive care treatment, treatment in hospital, transfer to hospital if a diagnosis is not possible at the RACF, treatment aiming to prolong life at the RACF, comfort care.	

Justification of these anticipatory decisions :

Emergency Contacts

HEALTH CARE PROXY

Name ______________________ Relation ____________________

Tel. 1 ______________________ Tel. 2 ______________________

Named health care proxy according to (select only one response):
□ Named in the resident's Advanced Directive
□ Named as an Enduring Power of Attorney (medical decisions/ general)
□ According to the legal hierarchy in article 378 of the Swiss civil code

TREATING PHYSICIAN

Name : ____________________ Practice Tel : ____________________

Practice : ____________________ Mobile Tel :__________________

Advance Care Planning by proxy Facilitator

Name ____________________ Tel. 1 ____________________

Position : ____________________

Back up CONTACT : if the health care proxy is unavailable (without legal decision making status)

Name ______________________ Relation ____________________

Tel. 1 ______________________ Tel. 2 ______________________

SIGNATURES

Health Care Proxy

Name: _____________________ Signature:_____________________ Date:

Treating Physician

Name: _______________________ Signature: ___________________ Date:

ACP by proxy facilitator

Name: ________________________Signature: ___________________ Date:

Facilitator Self-efficacy Scale

Health professional self-efficacy in providing care that is coherent with resident wishes scale

To what extent are you confident that you are providing care that is consistent with residents' wishes?

1	5	10
Not at all confident	Moderately confident	Very confident

Health care proxy post-intervention semi-structured interview guide

1. Can you tell me about the discussions about the values and wishes for your (relation)'s future care?

2. Generally speaking, what did you think of the process?
   - Were there any parts that you found easy?
   - Were there any parts that you found difficult?
   - Have these discussions been relevant for you? In which way?
   - Has this process helped you in any way in your role as a health care proxy?
     - In what way?

3. Can you tell me about how the discussions were conducted?
   - Are there any aspects of these discussions that you particularly appreciated?
   - Were there any parts of these discussions that you did not find useful/necessary?
   - Were there any aspects of the facilitators skills that you particularly appreciated?
   - Are there any skills that the facilitator could further develop to improve these discussions?

4. Can you tell me about the second discussion, during which you met the treating physician and made anticipatory decisions on your (relation)'s behalf?
   - What did you think of this discussion?
   - What role did the GP play?
   - Do you feel you had all the information you needed to make these decisions?

   Did you feel supported in making this decision?

   What factors contributed to this?

5. What did you think about documenting the anticipatory decisions?
   - Do you think this documentation will be useful for you in the future?

6. Generally speaking, do you have any suggestions for improving this process in the future?

7. Would you like to make any other remarks or comments about the intervention, how it went and how it could be improved in the future?

Health professional post-intervention semi-structured interview guide

8. Generally speaking, what do you think of the advance care planning by proxy intervention?
    - How do you think the process went?

9. Can you talk to me about your experience about conducting this ACP-bp process?
    - Were there any parts of the process that were easy to conduct?
    - Were there any parts of the process that were difficult to conduct?
    - What skills do you think are particularly important for leading these discussions?
    - What skills do you feel you could develop to improve discussions about advance care planning by proxy?

10. How do you perceive the health care proxies acceptance of this process?
    - Were there any parts that they seemed to like?
    - Were there any parts that seemed difficult?
        - o Do you have any suggestions for overcoming these difficulties?
    - Were there factors that changed their acceptance (were some health care proxies more open to the process than others?
    - In your opinion, did the therapeutic representatives find the intervention useful? In what way? If not, why not?

11. In your opinion, was the process a success?
    - Why/why not?
    - What factors have contributed to its success?
    - What specific parts of the intervention worked well?
    - Which parts of the intervention did not work well?
    - Do you have any suggestions about how we can improve the process in the future?

12. Has this advance care planning by proxy intervention brought about any changes in the RACF?
    - What types of changes? What were the reasons for the changes?

13. Would you like to make any remarks or comments about the intervention, how it went and how it could be improved in the future?
